# Deferral of Treatment for Small Choroidal Melanoma and the Risk of Metastasis: An Investigation Using the Liverpool Uveal Melanoma Prognosticator Online (LUMPO)

**DOI:** 10.3390/cancers16081607

**Published:** 2024-04-22

**Authors:** Bertil Damato, Antonio Eleuteri, Azzam Taktak, Rumana Hussain, Maria Fili, Gustav Stålhammar, Heinrich Heimann, Sarah E. Coupland

**Affiliations:** 1 Ocular Oncology Service, St. Erik Eye Hospital, 17164 Stockholm, Sweden; maria.fili@regionstockholm.se (M.F.); gustav.stalhammar@ki.se (G.S.); 2 Department of Clinical Neuroscience, Division of Eye and Vision, Karolinska Institutet, 17177 Solna, Sweden; 3 Molecular and Clinical Cancer Medicine, University of Liverpool, Liverpool L7 8XT, UK; 4 Ocular Oncology Service, Moorfields Eye Hospital, London EC1V 2PD, UK; 5 NHS Digital Services, Liverpool University Hospitals NHS Foundation Trust, Kilby House, Liverpool Innovation Park, Liverpool L7 9NJ, UK; antonio.eleuteri@liverpoolft.nhs.uk; 6 Clinical Engineering, University Hospitals Bristol and Weston NHS Foundation Trust, Bristol BS2 8HW, UK; azzam.taktak@uhbw.nhs.uk; 7 Ocular Oncology Service, Liverpool University Hospitals NHS Foundation Trust, Liverpool L7 8XP, UK; rumana.hussain@liverpoolft.nhs.uk (R.H.); heinrich.heimann@gmail.com (H.H.); 8 Department of Eye and Vision Science, University of Liverpool, Liverpool L7 8XT, UK; s.e.coupland@liverpool.ac.uk

**Keywords:** choroidal melanoma, treatment, metastatic death, non-metastatic death, monosomy 3

## Abstract

**Simple Summary:**

Choroidal melanomas are treated in the hope of preventing metastatic death; however, such treatment often causes severe visual loss. There is debate as to whether the treatment of small tumors can be deferred until tumor growth is observed. Using the Liverpool Uveal Melanoma Prognosticator Online v.3 (LUMPO3), this study provides an indication of change in the 15-year absolute risk of metastatic death, according to whether the tumor is treated immediately or after 4 or 12 months, when growth is observed. It considers tumor growth rate, dimensions, laboratory indicators of metastasis risk, prevalence of monosomy 3, patient’s age, and sex. Our study suggests that deferring treatment until growth is observed is associated with minimal, if any, increase in the risk of metastatic death with usual tumor growth rates of up to 40% per year. It would seem reasonable to delay treatment until tumor growth is documented, especially if this is likely to cause visual loss.

**Abstract:**

Background: We estimated metastatic-death risk when the treatment of small choroidal melanomas is deferred until growth is observed. Methods: In 24 patients with choroidal melanoma (median diameter 5.85 mm), the exponential growth rate estimated by a mixed-effects model was 4.3% per year. Using the Liverpool Uveal Melanoma Prognosticator Online v.3 (LUMPO3), we measured changes in 15-year metastatic and non-metastatic death risks according to whether the tumor is treated immediately or after observing growth 4 or 12 months later, considering age, sex, and metastasis predictors. Results: In 40-year-old females with 10 mm, disomy 3 and monosomy 3 choroidal melanomas (prevalence 16%), the 15-year absolute risks of metastatic death are 4.2% and 76.6%, respectively, increasing after a 4-month delay by 0.0% and 0.2% and by 3.0% and 2.3% with tumor growth rates of 5.0% and 20.0%, respectively. With 12-month delays, these risks increase by 0.0% and 0.5% and by 1.0% and 7.1%, respectively. Increases in metastatic-death risk are less with smaller tumors and with a higher risk of non-metastatic death. Conclusions: Deferring treatment of choroidal melanomas until documentation of growth may delay iatrogenic visual loss by months or years and is associated with minimal increase in metastatic mortality, at least with small tumors with usual growth rates of up to 40% per year.

## 1. Introduction

Uveal melanomas can cause visual loss, a painful eye, and metastatic disease, which is usually fatal within a year of the onset of symptoms [1,2]. Risk factors for metastatic death include male sex; older age; larger tumor diameter; greater tumor height; ciliary body involvement; extraocular spread; epithelioid melanoma cell type; extravascular matrix patterns such as closed loops; greater tumor volume; chromosome 3 loss (i.e., monosomy 3); chromosome 8q gain; and several other factors, such as class 2 gene expression profile, BAP1 loss, PRAME expression, and *SF3B1* mutation [2,3,4,5,6,7,8,9,10,11]. It has been suggested that metastatic spread can commence when the tumor is as small as 3 mm in basal diameter [12].

Ocular treatment is aimed at preventing metastatic disease, if possible, conserving the eye and useful vision [2,13]. There is much debate regarding the management of patients with small choroidal melanomas [14]. Some authors advocate immediate treatment to maximize any opportunities for preventing metastatic disease and death, even if the treatment is likely to cause severe visual loss [15,16,17,18]. Others defer treatment until growth is documented, to conserve useful vision as long as possible, because they consider this approach to have minimal or no risk of increasing metastatic mortality [19,20,21,22]. Randomized clinical trials with adequate statistical power are prevented by the need for large numbers of patients and long follow-up. This is because, with small choroidal melanomas, metastatic disease is rare and tends to develop after a long period, by which time many patients have died of unrelated disease or been lost to follow up.

The Liverpool Uveal Melanoma Prognosticator Online version 3 (LUMPO3) has been developed to estimate the absolute risk of metastatic death in patients with choroidal melanoma by multivariable analysis of anatomic, histologic, and genetic predictors, also taking age and sex into account [23]. This prognostic tool has been validated by a multicenter study [24].

The aim of this study was to determine change in metastatic and non-metastatic mortality predicted by LUMPO3 if treatment of choroidal melanomas is deferred by 4 or 12 months until tumor growth is detected. To do this, we fitted a growth model of tumor diameter using data from a cohort of 24 patients whose treatment was deferred by a minimum of 4 months. We also estimated growth of tumor height indirectly by use of a median regression model, which determines the association between tumor diameter and tumor height. Tumor diameter and tumor height were then used, together with other predictors, as inputs to LUMPO3 to prognosticate the absolute 15-year risks of metastatic and non-metastatic death.

## 2. Materials and Methods

### 2.1. Patients

Patients attending the ocular oncology service at St. Erik Eye Hospital between May 2022 and September 2023 were included in this study if they were diagnosed as having a choroidal melanoma not exceeding 11 mm in diameter, and if treatment was deferred by at least four months. To estimate the growth rate of tumor diameter by mixed effects modelling, one patient was excluded because of extremely rapid growth after declining treatment; the exponential growth rate for this subject was 74% per year, while the second largest growth rate was only 40% per year. Such leverage points can have an overriding influence on the fitted model.

### 2.2. Methods

The electronic medical records were reviewed together with color or pseudo-color fundus photographs (Optos plc., Dunfermline, UK), autofluorescence images (Optos plc., Dunfermline, UK), optical coherence tomography (OCT) (Topcon, Tokyo, Japan), and ultrasonography B-scans (Quantel Medical, Cournon d’Auvergne, France). The following data were documented: age, sex, affected eye, location of anterior and posterior tumor margins, tumor extension through Bruch’s membrane, lipofuscin accumulation, largest basal tumor diameter, tumor thickness, documented growth, and extent of subretinal fluid. Basal tumor dimensions were measured using calipers in Optos images, or OCT if this revealed lateral tumor spread not visible in the Optos image. When these methods were not possible, the horizontal disc diameter was utilized as a measure, assuming this to be 1.5 mm. Tumor thickness measurements were retrieved from OCT images, ultrasonography B-scans, or patient’s electronic medical records. Orange pigment was assessed by reviewing fundus autofluorescence images or, if these were not available, color or pseudo-color fundus photographs and OCT images. Subretinal fluid was assumed to be absent if OCT imaging was not available and if this feature was not documented in the medical records. Tumors were given MOLES scores as previously described [25].

### 2.3. Mortality Estimation

Absolute risk of metastatic and non-metastatic mortality within 15 years of presentation were estimated in hypothetical 40-, 60-, and 70-year-old males and females with tumors ranging from 4 to 10 mm in diameter, and according to whether laboratory investigations reveal features associated with increased metastatic death risk. Laboratory risk factors are categorized as (a) ‘low’ if epithelioid cells, closed loops, monosomy 3, and chromosome 8q gain are all absent, and if the mitotic count per 40 high-power fields is 0 or 1 and (b) ‘high’ if epithelioid cells, closed loops, monosomy 3, and chromosome 8q gain are all present, and if the mitotic count per 40 high-power fields is more than 7. The absolute risk of metastatic death was calculated according to whether treatment was immediate or deferred by four or twelve months, with attendant tumor growth of diameter and height inferred by use of two ad-hoc models.

Metastatic mortality for this study was calculated using the offline version of the Liverpool Uveal Melanoma Prognosticator Online, version 3 (LUMPO3). This estimates the metastatic mortality and non-metastatic mortality until 15 years after ocular treatment by multivariable analysis of anatomic metastasis predictors (i.e., largest basal tumor diameter, tumor height, ciliary body involvement, and extraocular spread); histologic predictors (i.e., melanoma cytomorphology, closed loops, mitotic count per 40 high-power fields); and genetic predictors (i.e., chromosome 3 loss and chromosome 8q gain), also taking account of age and sex to avoid bias from competing risks. LUMPO3 is implemented as a multi-state Markov model with underlying Cox state transition intensities to model the competing risks of metastatic and non-metastatic death. This tool was developed with data from patients treated for choroidal melanoma at the Liverpool Ocular Oncology Centre and residing in England, Scotland, and Wales. The cohort comprised 4161 patients (51% male) with a median age of 62.5 years. The tumors had a median diameter of 12.4 mm and a median thickness of 5.0 mm, with epithelioid cells in 58% of 2187 tumors, chromosome 3 loss in 45% of 602 tumors, and chromosome 8q gain in 45% of 602 tumors tested. Patients were flagged prospectively at the National Health Service Cancer Registry, which provided notification of date and cause of death, which was metastatic and non-metastatic in 893 and 772 patients, respectively. Values for missing metastasis predictors were estimated by modeling their relationships with all other available data, using the Alternating Conditional Expectations algorithm coupled with an approximate Bayesian bootstrap. Patient consent was waived due to our use of fundus images that had been registered for clinical diagnostic and prognostic purposes, with no additional interventions for the purposes of this study, and because no analyses of biological tissues were performed for this project. The study was conducted according to the guidelines of the Declaration of Helsinki and approved by the Swedish Ethical Review Authority (reference 2022 06210-02, 9 December 2022).

### 2.4. Tumor Growth Rate Estimation

The tumor diameter growth rate was estimated from the cohort of 24 patients by fitting a linear mixed effects model to the longitudinal observations of the natural logarithm of tumor diameter [26]. It can be shown that estimation of the model’s slope coefficient corresponds to estimation of exponential growth rate, which has been described in literature as a biologically plausible model [27]. Mixed effects models have been shown to have high power and correct type I error rates [28]. The estimated growth rate was 4.3% per year (1.9%, 6.7%), *t*(23) = 3.74, *p* = 0.001. There was no evidence of a random effect of growth rate, but estimated standard deviation of tumor diameter at presentation was 0.46 mm (0.33 mm, 0.64 mm). The estimated intra-class correlation was 0.71.

An exponential growth rate model was also fitted to the data reported by Jouhi et al. [29]; the estimated growth rate was 20% per year (11.7%, 27.6%), *t*(6) = 6.03, *p* = 0.001. There was no evidence of a random effect of growth rate; estimated standard deviation of tumor diameter at presentation was 0.26 mm (0.11 mm, 0.64 mm). The estimated intra-class correlation was 0.51.

Estimates of absolute risks of metastatic death and death due to other causes have been obtained for a set of simulated patients, with tumor diameter growth rates ranging from the estimated (approximate) expected 5% per year in the Swedish cohort, through expected 20% per year estimated from the Jouhi et al. cohort (i.e., the Finnish cohort) [29], up to the observed 40% per year (the largest in the Swedish cohort after removal of an outlier).

### 2.5. Chromosome 3 Prevalence Estimation

The prevalence of chromosome 3 loss as a function of tumor diameter, tumor thickness, patient age, and sex was estimated by fitting a logistic regression model [30]. The model was fitted with the same data used to fit the original LUMPO model, deploying the same multiple imputation procedure. The continuous predictors were nonlinearly expanded using restricted cubic splines of the 4th order. The Wald statistics for the predictors are: age ($\chi^{2}\left(3\right)=11,\;p=0.014$), sex ($\chi^{2}\left(1\right)=0.5,\;p=0.47$), tumor diameter ($\chi^{2}\left(3\right)=20,\;p=0.0001$), and tumor thickness ($\chi^{2}\left(3\right)=5.3,\;p=0.15$). The model’s AUC is 0.72, and the Brier score is 0.21.

### 2.6. Relationship between Tumor Diameter and Tumor Height Estimation

**Figure 1:**
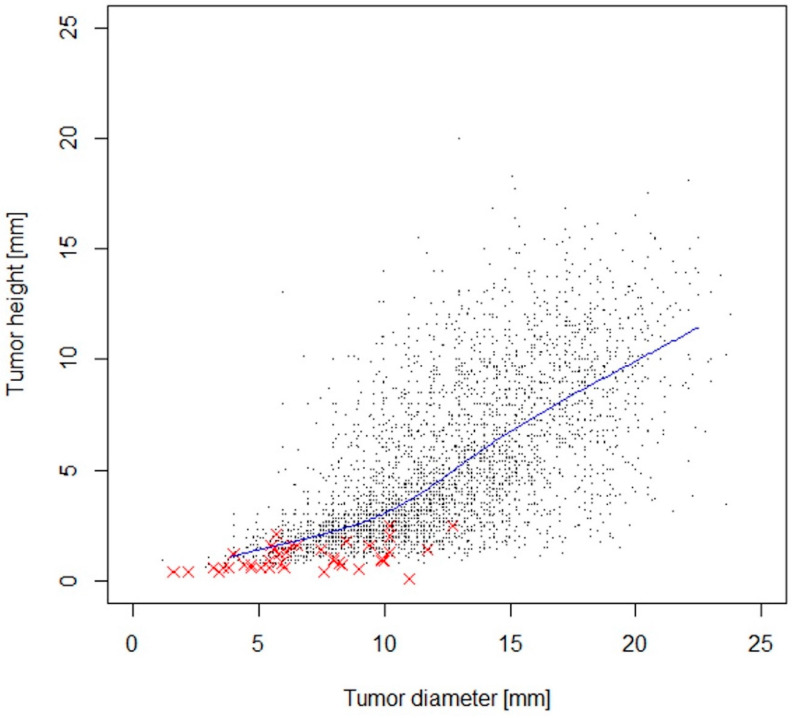
Scatter plot of tumor diameter vs tumor height. LUMPO3 cohort data (black dots) with Swedish cohort data (red crosses) and estimated median regression function (blue line).

A scatter plot of tumor diameter versus tumor height of LUMPO3 cohort data suggests a nonlinear association, with evidence of heteroskedasticity (i.e., the variance of tumor height increases as tumor diameter increases) (Figure 1). To avoid inefficiency of the regression estimates and biasedness of standard errors (and attendant tests) due to heteroskedasticity, a median regression model was fitted [31]; tumor diameter was nonlinearly expanded using restricted cubic splines of 4th order. The Wald statistic for the model is $\chi^{2}\left(3\right)=4343,\;p<0.0001$. The predictions of this model are used in the scenarios to indirectly estimate growth of tumor height corresponding to the growth of tumor diameter.

## 3. Results

### 3.1. Patients

The cohort comprised 24 patients (14 male, 10 female) with a mean age of 68 years (range, 46.2–81.9) (Table 1). Eighteen of these patients were first seen before May 2022, when they were managed by a different team of ocular oncologists.

The right eye was affected in 15 patients. The posterior tumor margin was located at disc and fovea (*n* = 3), fovea (*n* = 1), disc (*n* = 3), macula (*n* = 10), posterior choroid (*n* = 5) and equator (*n* = 2). The largest basal tumor diameter had a median of 5.85 mm (range, 1.6–11.0) and the tumor thickness had a median of 0.95 mm (range, 0.4–2.5).

The follow up had a median of 2.8 years (range, 0.3–11.1). The median increase in largest basal tumor diameter was 1.3 mm (range, 0.0–4.7). The median exponential growth rate was 3.6% (range, 0–40.2%).

### 3.2. Baseline Estimates

With immediate treatment, the 15-year risk of metastatic death ranges from 2.2% in 40-year-old females with a 4-mm tumor showing no high-risk laboratory features to 76.6% in 40-year-old females with a 10-mm tumor with high-risk laboratory findings. (Table 2) The predicted prevalence of monosomy 3 ranges from 12.9% in 40-year-old females with a 4-mm tumor to 31.2% in 70-year-old men with a 10-mm tumor (Table 2). The risk of non-metastatic death ranges from 1.7% in 40-year-old women with a high-risk, 10-mm tumor and a high-risk of metastatic death, to 50.4% in 70-year-old men with a low-risk, 4-mm tumor.

### 3.3. 4-Month Estimates

With disomy 3, low-risk choroidal melanomas up to 10 mm in diameter, a 4-month delay increases metastatic death risk by a maximum of 0.0% or 0.3% according to whether the growth rate is 5% per year (Table 3) or 20% per year (Table 4). With monosomy 3 melanomas, the maximum metastatic death risk increase is 0.3% (Table 3) or 2.3% (Table 4), respectively, according to these growth rates. Increase in metastatic death risk is highest in 40-year-old females with a 10-mm, monosomy-3 melanoma growing exceptionally fast (hypothesized 40% exponential growth) to 11.4 mm after only 4 months (Table S4); in such patients, the metastatic death risk increases by 5.1% from 76.6% (60.8%, 96.7%) to 81.7% (67.8%, 98.5%). The metastatic death risk is lower in patients with smaller tumors, slower tumor growth rates, spindle-cell morphology, no lethal genetic abnormalities, and/or higher risk of non-metastatic death, as in older patients, especially men. For example, at presentation, 60-year-old males with a 6-mm melanoma have 15-year, absolute metastatic and non-metastatic death risks of 2.9% (2.2%, 4.0%) and 25.1% (22.4%, 28.2%), respectively, with disomy-3 melanoma, and 62.6% (45.3%, 86.5%) and 13.7% (10.2%, 18.3%) absolute metastatic and non-metastatic death risks with monosomy-3 melanoma (Table S1) (prevalence is 23.5% (9.5%, 47.3%) in such patients (Table S5). After deferring treatment for 4 months, the risk of metastatic death increases by 0.0% and 0.1% with growth rates of 5% and 20%, respectively, if the tumor has low-risk features; with such growth rates, the increases in risk of metastatic death are 0.1 and 1.1%, respectively, if the tumor shows monosomy 3 and other metastasis predictors (Tables S1 and S3).

The greatest difference between monosomy-3 prevalence at presentation and that after a 4-month delay in treatment is 4.5% and this is in a 70-year-old man with a 10-mm tumor showing a 40% size increase per year (Table S4).

### 3.4. 12-Month Estimates

Estimates of change in absolute risk of metastatic and non-metastatic death if treatment is delayed by one year are provided according to whether the exponential tumor growth rate is 5% per year (Table 5) or 20% per year (Table 6). The greatest increase in risk of metastatic death after such delay is 15.4%, which occurs in a woman presenting at the age of 40 years with a high-risk, melanoma measuring 10.0 mm in diameter and 3.04 mm in thickness, with an exceptionally high growth rate of 40% per year, developing a diameter of 14.9 mm and a thickness of 6.68 mm (Table S4). The greatest increase in monosomy 3 prevalence is 21.0%, occurring in a 70-year-old male patient presenting with a 10.0 mm diameter tumor growing at a rate of 40% per year (Table S6). The greatest decrease in non-metastatic mortality is 7.8% and this is in a 70-year-old man with a 10-mm-diameter tumor showing high-risk laboratory features and a 40% per year growth rate (Table S4).

Supplementary Tables S1–S4 provide estimates of absolute 15-year risk of metastatic and non-metastatic death, with 95% confidence intervals, according to age, sex, tumor dimensions, laboratory indicators of metastatic death risk, tumor growth rate, and length of deferment of treatment. Supplementary Tables S5 and S6 estimate change in monosomy-3 prevalence with treatment deferral of 4 and 12 months, respectively.

## 4. Discussion

### 4.1. Purpose

The present study provides an approximate indication of any change in the 15-year risk of metastatic death according to whether the tumor is treated immediately or after 4 or 12 months if growth is observed, taking into account the tumor’s rate of growth, initial dimensions, presence or absence of laboratory indicators of metastasis risk, likelihood of these predictors being present, as indicated by known prevalence of monosomy 3, and the patient’s age and sex. Our results may help patients with a small, posterior choroidal melanoma to decide whether to (a) undergo treatment immediately, in the hope of preventing metastatic disease, or (b) defer treatment until tumor growth is documented, to conserve useful vision as long as possible. Shields et al. have shown that tumor growth within five years of observation occurs in only about 55% of choroidal melanomas with all five TFSOM features (i.e., thickness exceeding 2 mm, fluid under the retina, symptoms, orange pigment, and melanoma hollow on ultrasonography) [32].

### 4.2. Methods, Strengths and Weaknesses

We based our estimates of exponential tumor growth rate of diameter on data from a cohort of 24 patients whose treatment of choroidal melanoma was delayed by four months or more (Table 1) and it was found to be approximately 4%. This is similar to the results of Gass, whose data indicate a median growth rate of 4.7% per year (mean 8.5% per year, range 0% per year–38% per year) [33]. We would have preferred to base the estimates of growth on a larger cohort, but such patients are rare in our service because we have tended to treat all choroidal melanomas urgently, in accordance with current conventional practice. Although Jouhi et al. used exponential growth for tumor volume, they assumed a linear, rather than exponential increase in largest basal tumor diameter when they reported a sample estimate of linear increase in diameter of 34% per year [29]. They based this on the median of the linear growth rates estimated for each patient, rather than a model. We therefore fitted an exponential growth rate model to their data, which resulted in an estimated exponential growth rate of 20% per year. Data reported by Augsburger et al. indicate the growth rate to have a median of 81% per year (mean 110% per year, range 7.6%/year–374% per year) and 24% per year (mean 41% per year, range 6.2% per year–162% per year) in tumors with and without epithelioid melanoma cells, respectively [34]. We cannot explain the variation in growth rates between the different cohorts. Further studies with data from other centers would be interesting. Although our tables only estimate risks with growth rates up to 40%, clinicians and patients need to accept that if treatment is deferred the tumor may be found to have a much higher growth rate. 

We estimated how much tumor dimensions would increase after 4 or 12 months according to these growth rates. The tumor height-to-diameter ratios estimated using the original LUMPO cohort were greater than those seen in our Swedish cohort (Figure 1). This is perhaps because tumor height was measured in Stockholm using optical coherence tomography whereas in Liverpool this measurement was obtained with ultrasonography, which is known to give higher values [35].

To our knowledge, no other studies have addressed the question of deferred treatment using models of growth and models of metastatic death. We previously reported LUMPO3 to have C-index of 0.861 (95% confidence interval (0.84, 0.88)) and calibration curves indicate that this tool provides a reliable, personalized metastatic death prognostication [23]. Further, the LUMPO3 estimates that are provided by this study are similar to those of previous investigations with respect to actuarial mortality rates as well as associations between metastatic mortality and older age, male sex, larger tumor size, higher histological grade of malignancy, chromosome 3 loss, and chromosome 8q gain [4,5,36]. Furthermore, our study found that the prevalence of chromosome 3 loss is higher in older patients, as reported previously [36,37]. For example, in patients with a 10 mm diameter tumor, the prevalence of chromosome 3 loss increases from 15.8% to 28.4% in 40-year-old and 70-year-old females, respectively (Table 2). We also found that chromosome 3 loss is more common in males than females, as reported previously [36,37]. For example, this genetic aberration is present in 28.4% and 31.2% 70-year-old females and males with a 10-mm choroidal melanoma, respectively (Table 2). These results suggest that it would be reasonable to use LUMPO3 when counseling patients about immediate versus deferred treatment according to a wide variety of anatomic, histologic, and genetic combinations.

LUMPO3 was developed to estimate the absolute risk of death after immediate treatment of choroidal melanoma and not to estimate change in risk of metastatic death arising from deferred treatment. The LUMPO3 model and our model of monosomy-3 prevalence do not take account of tumor growth rate (or, more generally, of the history of tumor growth). For example, Table 2 indicates a 0.3% increase in monosomy-3 prevalence and a 1% increase in the 15-year absolute risk of metastatic death in a 60-year-old females whose largest basal tumor diameter has increased from 6.0 mm to 6.41 mm in 4 months (i.e., exponential growth rate, 20% per year); however, if monosomy 3 prevalence and the metastatic death risk depended on the history of tumor growth, then the estimates associated with a 6.41 mm tumor would be lower if its growth rate is 5% per year than if it is 40% per year. There is scope for further studies correlating tumor growth history with metastatic mortality and with prevalence of genetic predictors. Until the results of such studies become available, estimates provided in this study should be regarded as a working hypothesis.

As LUMPO3 estimates are not based on sequential measurements of tumor dimensions, it would not be correct to infer that it is the delay in treatment and the tumor growth that increase the 15-year risk of metastatic death. It may do so in some patients, for example, if a tumor transforms from disomy 3 to monosomy 3, as suggested by the higher prevalence of chromosome 3 loss in older patients, as mentioned above, and as seen in a patient whose slow-growing tumor was not immediately treated [38]. Conversely, the high incidence of metastatic death in patients with small, high-risk melanomas (e.g., 53.3% in 40-year-old females with a 4-mm tumor treated immediately (Table 2)) indicates that many tumors have already metastasized by the time of presentation so that tumor growth during the treatment delay only reflects the probability of lethal aberrations being present and hence the probability that the tumor has already metastasized by the time the patient first presents. This hypothesis is supported by Augsburger’s study showing that uveal melanomas with a greater metastatic potential, as evidenced by the presence of epithelioid cells, have a higher growth rate than spindle-cell melanomas [34].

An advantage of using LUMPO3 is that it estimates risk of metastatic and non-metastatic death even in patients with rare combinations of risk factors and this is because prognostications are based on extrapolations when anatomic, histologic, and/or genetic data are missing. Some authors may question this approach; however, uncertainty in the predictions is reflected by the width of the confidence intervals in the tables.

It would have been ideal if malignancy in these patients had been confirmed by histologic and genetic analysis, but, like most other centers, we do not routinely perform biopsy of such small tumors. Instead, we have based our diagnosis on the MOLES system, which indicates the likelihood of malignancy according to mushroom shape, orange pigment, large size, enlargement, and subretinal fluid [25]. MOLES scores have been shown to correlate with clinician’s diagnosis of melanoma versus nevus.

A weakness of this study is that the genetic data used to train LUMPO3, includes results from fluorescence in situ hybridization (FISH), which was used in the early years of this study, and which has since been superseded by methods such as multiplex ligation-dependent probe amplification (MLPA) and microsatellite analysis (MSA). This factor accounts for the estimated metastatic mortality in patients with low-risk laboratory features, including disomy 3.

The risks of non-metastatic death estimated by LUMPO3 are slightly higher than those reported in current 2021 UK census data, and this is possibly because the certified cause of death may have been incorrect in some patients, with metastatic death misdiagnosed as death from another disease.

### 4.3. Results

The metastatic-death risk is inversely related to the risk of non-metastatic death (because patients who die of other causes cannot then die a second time, of melanoma). For example, the 15-year risk of metastatic death in a 40-year-old woman with a high-risk, 10-mm tumor is 76.6%, compared to 73.1% in a 70-year-old woman with an identical tumor. This is because the older woman has a 17.3% risk of non-metastatic death, compared to 1.7% in the 40-year-old woman (Table 2). Conversely, in a 60-year-old woman with an 8-mm tumor, the 15-year risk of non-metastatic death is 19.9% if the tumor biopsy shows no metastasis predictors, but only 9.8% if these features are present (Table 2).

Assuming that there is no change in histologic and genetic features (and therefore no increase in the tumor’s metastatic potential) during the treatment delay, the 15-year risk of metastatic death from the time of presentation is unchanged if the increase in metastatic death risk according to tumor growth is balanced by the decrease in metastatic death risk because the patient has survived the period of treatment delay. In some cases, the metastatic death risk may even diminish, as in a 60-year-old male presenting with a 4-mm, high-risk tumor in whom this risk reduces from 55.2% to 54.3% (i.e., −0.9%) if he survives the first year after presentation, during which time the tumor has increased to 4.21 mm (Table 5b). This is because the less time that remains before the age of 75 years, the less the risk of metastatic death before this age becomes.

In this study, the rate of tumor growth seems to be associated with risk of metastatic death within 15 years of presentation. For example, if a 40-year-old male presents with a high-risk, 10 mm choroidal melanoma, the risk of metastatic death before the age of 55 is 78.9% if the tumor has a 10% growth rate so that its diameter is 10.3 mm after 4 months (Table S2) and slightly less, at 78.5%, if the tumor has a 5% growth rate so that its diameter increases to 10.5 mm after 12 months (Table S1). This is probably because the growth rate indicates the likelihood of the melanoma having metastatic potential, as suggested by the study by Augsburger et al., showing that uveal melanomas with a higher growth rate are more likely to have epithelioid cells, and therefore, a greater metastatic potential [34]. If tumor biopsy reveals no histologic and genetic predictors of metastasis, the rate of tumor growth is less relevant, except that a high growth rate suggests a failure to detect lethal abnormalities.

### 4.4. Clinical Implications

The decision whether to treat or monitor the tumor must take account of the patient’s needs, wishes, and fears, which are influenced by vision in the fellow eye, occupation, age, sex, and other factors. In the absence of definitive data from randomized clinical trials, decisions on patient management are inevitably influenced by the intuitive beliefs of the clinicians involved in the patient’s care, which may result in a lack of consensus even amongst the authors of this article. Clinical significance is more relevant to patient care than statistical significance because of all these considerations, although uncertainty of the estimates can be inferred from confidence intervals. In any case, it is essential for clinicians to ensure that patients are fully informed of all risks and benefits, both of immediate treatment and of deferring treatment. Understandably, patients and their relatives become highly dissatisfied with the care they received if, when an adverse outcome develops, they discover that they had been deprived of the opportunity to make an informed decision on treatment. It is also important to explicitly document in the medical records that informed consent for the selected management has been obtained. Patients tend to forget all that they are told at a clinical consultation so that it is helpful to provide them with an audio recording of the discussion.

Choroidal melanocytic tumors exceeding 6 mm in diameter are usually treated without delay if they show clinical features suggesting malignancy (e.g., a MOLES score of 3 or more). With tumors up to 6 mm in diameter, deferring treatment by 4 months is not associated with more than a 1% increase in the 15-year risk of metastatic death except in patients whose tumor has a growth rate of 40% per year, in whom this risk can be as high as 2.8% in 40-year-old females with a high-risk tumor. Some tumors have a growth rate that is much higher than 40% per year, as mentioned above. This possibility therefore needs to be considered when deciding whether to defer treatment. For example, the patient can be reviewed less than 4 months after the initial assessment or biopsy can be undertaken.

If biopsy is performed and if this reveals spindle-cell morphology and no lethal genetic aberrations, the greatest increase in 15-year risk of metastatic death after a 4-month delay is 0.8%, which occurs in 60-year males presenting with a 10 mm tumor, in whom the 15-year metastatic death risk after immediate treatment would be 4.5% (Table S4).

If, however, biopsy shows the tumor to have high-risk features (i.e., epithelioid, or even anaplastic cell morphology and lethal genetic aberrations), the greatest increase in this risk after a 4-month delay is 4.9%, which occurs in 40-year-old males presenting with a 10 mm tumor (Table S4). At the initial visit, such patients already have a 78% risk of metastatic death by the age of 55 years, so it would be prudent not to defer treatment in case any metastatic disease is ever attributed to this delay, whatever the possible increase in metastatic death risk.

These estimates suggest that, ideally, all patients with a small choroidal melanoma should be offered biopsy when deciding whether to defer treatment until growth is observed, and this is what is done at the Liverpool Ocular Oncology Centre [39]. Biopsy would provide greater reassurance if genetic predictors of metastasis are not found, especially if findings such as chromosome 6p gain and an *EIF1AX* mutation suggest that such lethal mutations are less likely to develop. As mentioned, if biopsy indicates a high risk of metastasis, immediate treatment may be advised as the patient may regret delaying treatment, blaming this for metastatic disease if such an adverse outcome were to occur. If aggressive malignancy has been confirmed by biopsy, there is no longer any need to wait for growth to be documented, especially as this is most likely to happen.

When biopsy is not performed, it may be useful to consider the probability of the tumor having lethal genetic aberrations, which ranges from 13% (95% CI 3–42%) in 40-year-old females with a 4-mm tumor to 31% (95% CI 2–45%) in 70-year-old males with a 10-mm tumor (Table S5). Depending on the patient’s attitude to the risks and benefits of deferred treatment, the tumor could be treated immediately, or the patient could be reviewed sooner than four months after the initial visit.

### 4.5. Further Research

In some centers, small melanocytic choroidal tumors of uncertain malignancy are undergoing genetic analysis thanks to advances in biopsy technique and the development of sensitive genetic methods, such as next generation sequencing. Progress is also occurring in the analysis of genetic abnormalities in aqueous and blood samples [40,41,42]. These advances should in time enable more robust data to be obtained, which will allow risk of deferring treatment to be defined more accurately.

We aim to upgrade LUMPO3 as more data become available. The current online version limits prognostication to 10 years of follow up (although the underlying offline model used in this study allows prognostication to 15 years. The model may also be improved by re-training it with updated patient data and including other predictors, such as BAP1 and PRAME protein expression, mutations in *BAP1*, *EIF1AX*, and *SF3B1*, and number of copies of chromosome 8q (as opposed to the current binary categorization).

As mentioned, it would be useful to correlate tumor growth history with prevalence of genetic and histologic metastasis predictors, as well as with metastatic mortality; however, this would require large numbers of patients monitored for many years.

There is scope for assessing the psychological risks and benefits of deferring treatment as patients may intuitively believe that such procrastination is dangerous. It would also be useful to investigate whether well-intentioned, immediate treatment inadvertently harms patients by limiting their activities or increasing the risk of accidents because of visual loss.

## 5. Conclusions

The present study supports the conclusions of some previous authors that deferring treatment until growth is documented is associated with minimal if any increase in absolute risk of metastatic death, at least in tumors up to 6 mm in diameter with growth rates of up to 40% per year. This approach may enable some patients to retain useful vision for months or years, with some never needing to undergo any treatment at all.

## Figures and Tables

**Table 1:** Age, sex, surveillance duration, initial and final tumor dimensions, and rate of exponential growth in a cohort of patients with choroidal melanoma in Sweden whose treatment was deferred by more than 4 months.

Case	Age (Yrs)	Sex	Initial Largest Basal Tumor Diameter (mm)	Initial Tumor Height (mm)	Surveillance Duration (Yrs)	Final Largest Basal Tumor Diameter (mm)	Final Tumor Height (mm)	Exponential Growth Rate (%/yr)
1	66.6	Female	5.8	1.2	0.5	6.1	1.3	10.1
2	65.3	Male	4.7	0.6	0.8	4.7	0.7	0
3	74.4	Female	10.2	1.3	1	11.7	1.4	13.3
4	79.3	Male	5.5	1.6	3.1	10.2	2	19.7
5	60.9	Male	7.6	0.4	11.1	9	0.5	1.5
6	62.2	Male	2		7.8	3.8	0.6	8.3
7	65.2	Male	9.9	0.9	0.7	10	0.9	1.5
8	63.2	Male	3.4	0.4	10.4	4.4	0.7	2.5
9	81.9	Male	6	0.6	1.2	6	0.6	0
10	77	Male	9.9	0.9	0.3	9.9	1	0
11	62.7	Male	6.5	1.6	0.9	9.4	1.6	40.2
12	80.3	Female	1.6	0.4	4.4	5.4	0.6	27.4
13	51.8	Female	8	1	0.3	8	0.9	0
14	80.7	Male	5.6	1.4	2.4	6.3	1.6	4.8
15	57.4	Female	6.4		7.9	7.5	1.4	2
16	71.4	Female	11		1.2	11	0.1	0
17	79.2	Male	10.2	2.5	9	12.7	2.5	2.4
18	73.4	Female	2.1		4	3.6	0.6	13.6
19	46.2	Male	2		1.6	3.2	0.6	28.8
20	73.3	Male	2.4		4.2	5.1	0.6	18.1
21	77.4	Female	5.9	0.8	11	8.5	1.8	3.3
22	66.1	Female	4	1.2	9	5.7	2.1	3.9
23	58.7	Female	5.4		3.8	5.4	0.9	0
24	62.4	Male	6.5		2.3	8.3	0.7	10.6

**Table 2:** Prevalence of monosomy 3 and 15-year absolute risk of metastatic and non-metastatic death according to age, sex, tumor dimensions, and laboratory indicators of metastatic death risk at baseline. (a) Females, (b) Males.

Tumor Size	Age (yrs)	M3 Prevalence	Lab Risk Factors	Absolute 15-Year Risk	
				Metastatic Death	Non-Metastatic Death
(a)					
**Diam. 4.0 mm** **Ht 1.12 mm**	**40**	0.129	**low**	0.022	0.037
			**high**	0.533	0.024
	**60**	0.205	**low**	0.023	0.201
			**high**	0.545	0.124
	**70**	0.238	**low**	0.022	0.418
			**high**	0.517	0.257
**Diam. 6.0 mm** **Ht. 1.66 mm**	**40**	0.134	**low**	0.027	0.037
			**high**	0.61	0.022
	**60**	0.212	**low**	0.029	0.2
			**high**	0.621	0.111
	**70**	0.246	**low**	0.027	0.417
			**high**	0.589	0.231
**Diam. 8.0 mm** **Ht. 2.23 mm**	**40**	0.139	**low**	0.034	0.037
			**high**	0.688	0.019
	**60**	0.220	**low**	0.035	0.199
			**high**	0.696	0.098
	**70**	0.260	**low**	0.034	0.415
			**high**	0.661	0.203
**Diam. 10.0 mm ** **Ht. 3.04 mm**	**40**	0.158	**low**	0.042	0.037
			**high**	0.766	0.017
	**60**	0.160	**low**	0.044	0.198
			**high**	0.77	0.083
	**70**	0.284	**low**	0.042	0.412
			**high**	0.731	0.173
(b)					
**Diam. 4.0 mm** **Ht 1.12 mm**	**40**	0.140	**low**	0.023	0.048
			**high**	0.548	0.03
	**60**	0.228	**low**	0.024	0.252
			**high**	0.552	0.153
	**70**	0.263	**low**	0.022	0.504
			**high**	0.51	0.307
**Diam. 6.0 mm** **Ht. 1.66 mm**	**40**	0.150	**low**	0.028	0.048
			**high**	0.626	0.027
	**60**	0.235	**low**	0.029	0.251
			**high**	0.626	0.137
	**70**	0.271	**low**	0.027	0.503
			**high**	0.581	0.275
**Diam. 8.0 mm** **Ht. 2.23 mm**	**40**	0.156	**low**	0.035	0.048
			**high**	0.704	0.024
	**60**	0.244	**low**	0.036	0.25
			**high**	0.7	0.12
	**70**	0.281	**low**	0.033	0.5
			**high**	0.651	0.242
**Diam. 10.0 mm ** **Ht. 3.04 mm**	**40**	0.176	**low**	0.044	0.048
			**high**	0.78	0.021
	**60**	0.272	**low**	0.045	0.248
			**high**	0.772	0.102
	**70**	0.312	**low**	0.042	0.497
			**high**	0.72	0.206

**Table 3:** Change in absolute 15-year risk of metastatic and non-metastatic death if exponential tumor growth rate is 5% per year and if treatment is deferred by 4 months. (a) Females, (b) Males.

Tumor Size	Age (yrs)	M3 Prevalence	Lab Risk Factors	Absolute 15-Year Risk (from Baseline)	
				Metastatic Death	Non-Metastatic Death
(a)					
**Diam. 4.07 mm** **Ht. 1.14 mm**	**40.3**	0.001	**low**	0.000	0.000
			**high**	−0.002	0.000
	**60.3**	0.002	**low**	0.000	−0.004
			**high**	−0.002	−0.001
	**70.3**	0.001	**low**	0.000	−0.006
			**high**	−0.002	−0.001
**Diam. 6.10 mm ** **Ht. 1.69 mm**	**40.3**	0.001	**low**	0.000	−0.001
			**high**	−0.001	0.000
	**60.3**	0.002	**low**	0.000	−0.004
			**high**	−0.001	−0.001
	**70.3**	0.001	**low**	0.000	−0.007
			**high**	−0.002	−0.001
**Diam. 8.13 mm ** **Ht. 2.28 mm**	**40.3**	0.002	**low**	0.000	−0.001
			**high**	0.001	0.000
	**60.3**	0.003	**low**	0.000	−0.004
			**high**	0.000	−0.001
	**70.3**	0.001	**low**	0.000	−0.007
			**high**	−0.001	−0.001
**Diam. 10.20 mm ** **Ht. 3.13 mm**	**40.3**	0.004	**low**	0.000	−0.001
			**high**	0.002	−0.001
	**60.3**	0.005	**low**	0.000	−0.004
			**high**	0.001	−0.001
	**70.3**	0.005	**low**	0.000	−0.006
			**high**	0.002	−0.001
(b)					
**Diam. 4.07 mm** **Ht. 1.14 mm**	**40.3**	0.001	**low**	0.000	−0.001
			**high**	−0.002	0.000
	**60.3**	0.002	**low**	0.000	−0.004
			**high**	−0.003	−0.001
	**70.3**	0.001	**low**	0.000	−0.007
			**high**	−0.002	−0.001
**Diam. 6.10 mm ** **Ht. 1.69 mm**	**40.3**	0.001	**low**	0.000	−0.001
			**high**	−0.001	0.000
	**60.3**	0.002	**low**	0.000	−0.004
			**high**	−0.001	−0.001
	**70.3**	0.001	**low**	0.000	−0.008
			**high**	−0.002	−0.001
**Diam. 8.13 mm** **Ht. 2.28 mm**	**40.3**	0.002	**low**	0.000	−0.001
			**high**	0.000	0.000
	**60.3**	0.003	**low**	0.000	−0.004
			**high**	0.000	−0.001
	**70.3**	0.002	**low**	0.000	−0.007
			**high**	−0.001	−0.001
**Diam. 10.20 mm ** **Ht. 3.13 mm**	**40.3**	0.004	**low**	0.000	−0.001
			**high**	0.002	−0.001
	**60.3**	0.006	**low**	0.000	−0.004
			**high**	0.002	−0.001
	**70.3**	0.005	**low**	0.000	−0.007
			**high**	0.001	−0.001

**Table 4:** Change in absolute 15-year risk of metastatic and non-metastatic death if exponential tumor growth rate is 20% per year and if treatment is deferred by 4 months. (a) Females, (b) Males.

Tumor Size	Age (yrs)	M3 Prevalence	Lab Risk Factors	Absolute 15-Year Risk (from Baseline)	
				Metastatic Death	Non-Metastatic Death
(a)					
**Diam. 4.28 mm** **Ht. 1.19 mm**	**40.3**	0.002	**low**	0.000	0.000
			**high**	0.005	0.000
	**60.3**	0.002	**low**	0.001	−0.004
			**high**	0.006	−0.002
	**70.3**	0.001	**low**	0.000	−0.006
			**high**	0.005	−0.004
**Diam. 6.41 mm ** **Ht. 1.78 mm**	**40.3**	0.002	**low**	0.001	−0.001
			**high**	0.011	−0.001
	**60.3**	0.003	**low**	0.001	−0.004
			**high**	0.010	−0.003
	**70.3**	0.002	**low**	0.001	−0.007
			**high**	0.010	−0.006
**Diam. 8.55 mm ** **Ht. 2.42 mm**	**40.3**	0.004	**low**	0.001	−0.001
			**high**	0.017	0.000
	**60.3**	0.006	**low**	0.002	−0.004
			**high**	0.016	−0.004
	**70.3**	0.005	**low**	0.001	−0.007
			**high**	0.014	−0.007
**Diam. 10.70 mm ** **Ht. 3.44 mm**	**40.3**	0.014	**low**	0.003	−0.001
			**high**	0.023	−0.001
	**60.3**	0.019	**low**	0.003	−0.004
			**high**	0.020	−0.004
	**70.3**	0.019	**low**	0.003	−0.007
			**high**	0.020	−0.009
(b)					
**Diam. 4.28 mm** **Ht. 1.19 mm**	**40.3**	0.002	**low**	0.000	−0.001
			**high**	0.006	0.000
	**60.3**	0.003	**low**	0.000	−0.004
			**high**	0.005	−0.003
	**70.3**	0.002	**low**	0.000	−0.007
			**high**	0.005	−0.004
**Diam. 6.41 mm ** **Ht. 1.78 mm**	**40.3**	0.002	**low**	0.001	−0.001
			**high**	0.011	0.000
	**60.3**	0.003	**low**	0.001	−0.004
			**high**	0.011	−0.004
	**70.3**	0.002	**low**	0.001	−0.008
			**high**	0.009	−0.006
**Diam. 8.55 mm ** **Ht. 2.42 mm**	**40.3**	0.005	**low**	0.002	−0.001
			**high**	0.016	−0.001
	**60.3**	0.007	**low**	0.002	−0.005
			**high**	0.015	−0.005
	**70.3**	0.006	**low**	0.002	−0.007
			**high**	0.014	−0.009
**Diam. 10.70 mm ** **Ht. 3.44 mm**	**40.3**	0.015	**low**	0.003	−0.001
			**high**	0.022	−0.001
	**60.3**	0.020	**low**	0.003	−0.004
			**high**	0.020	−0.006
	**70.3**	0.020	**low**	0.002	−0.008
			**high**	0.019	−0.010

**Table 5:** Change in absolute 15-year risk of metastatic and non-metastatic death if exponential tumor growth rate is 5% per year and if treatment is deferred by 12 months. (a) Females, (b) Males.

Tumor Size	Age (yrs)	M3 Prevalence	Lab Risk Factors	Absolute 15-Year Risk (from Baseline)	
				Metastatic Death	Non-Metastatic Death
(a)					
**Diam. 4.21 mm** **Ht. 1.17 mm**	**41**	0.004	**low**	−0.001	0.000
			**high**	−0.009	0.000
	**61**	0.005	**low**	0.000	−0.002
			**high**	−0.008	0.001
	**71**	0.002	**low**	−0.001	−0.003
			**high**	−0.009	0.002
**Diam. 6.31 mm ** **Ht. 1.75 mm**	**41**	0.004	**low**	0.000	0.000
			**high**	−0.005	0.000
	**61**	0.006	**low**	−0.001	−0.002
			**high**	−0.005	0.001
	**71**	0.002	**low**	0.000	−0.004
			**high**	−0.006	0.001
**Diam. 8.41 mm ** **Ht. 2.37 mm**	**41**	0.006	**low**	0.000	0.000
			**high**	0.000	0.000
	**61**	0.008	**low**	0.000	−0.002
			**high**	−0.001	0.000
	**71**	0.005	**low**	0.000	−0.004
			**high**	−0.002	0.000
**Diam. 10.50 mm ** **Ht. 3.33 mm**	**41**	0.013	**low**	0.000	−0.001
			**high**	0.005	−0.001
	**61**	0.017	**low**	0.001	−0.002
			**high**	0.003	0.000
	**71**	0.015	**low**	0.000	−0.003
			**high**	0.003	−0.001
(b)					
**Diam. 4.21 mm** **Ht. 1.17 mm**	**41**	0.004	**low**	−0.001	0.000
			**high**	−0.008	0.001
	**61**	0.006	**low**	−0.001	−0.002
			**high**	−0.009	0.001
	**71**	0.002	**low**	−0.001	−0.003
			**high**	−0.008	0.003
**Diam. 6.31 mm ** **Ht. 1.75 mm**	**41**	0.004	**low**	0.000	0.000
			**high**	−0.005	0.001
	**61**	0.006	**low**	0.000	−0.002
			**high**	−0.005	0.001
	**71**	0.002	**low**	0.000	−0.004
			**high**	−0.006	0.002
**Diam. 8.41 mm ** **Ht. 2.37 mm**	**41**	0.006	**low**	0.000	−0.001
			**high**	−0.001	0.000
	**61**	0.008	**low**	0.000	−0.002
			**high**	−0.001	0.000
	**71**	0.005	**low**	0.000	−0.004
			**high**	−0.002	0.000
**Diam. 10.50 mm ** **Ht. 3.33 mm**	**41**	0.014	**low**	0.000	−0.001
			**high**	0.005	0.000
	**61**	0.019	**low**	0.001	−0.002
			**high**	0.004	−0.001
	**71**	0.015	**low**	0.000	−0.004
			**high**	0.002	−0.001

**Table 6:** Change in absolute 15-year risk of metastatic and non-metastatic death if exponential tumor growth rate is 20%/year and if treatment is deferred by 12 months. (a) Females, (b) Males.

Tumor Size	Age (yrs)	M3 Prevalence	Lab Risk Factors	Absolute 15-Year Risk (from Baseline)	
				Metastatic Death	Non-Metastatic Death
(a)					
**Diam. 4.89 mm** **Ht. 1.36 mm**	**41**	0.005	**low**	0.001	0.000
			**high**	0.017	−0.001
	**61**	0.008	**low**	0.001	−0.003
			**high**	0.017	−0.003
	**71**	0.004	**low**	0.001	−0.004
			**high**	0.015	−0.006
**Diam. 7.33 mm ** **Ht. 2.03 mm**	**41**	0.007	**low**	0.003	0.000
			**high**	0.035	−0.001
	**61**	0.009	**low**	0.002	−0.003
			**high**	0.033	−0.006
	**71**	0.006	**low**	0.003	−0.005
			**high**	0.031	−0.013
**Diam. 9.77 mm ** **Ht. 2.93 mm**	**41**	0.019	**low**	0.005	0.000
			**high**	0.054	−0.002
	**61**	0.027	**low**	0.006	−0.003
			**high**	0.050	−0.010
	**71**	0.025	**low**	0.005	−0.005
			**high**	0.047	−0.020
**Diam. 12.20 mm ** **Ht. 4.55 mm**	**41**	0.057	**low**	0.010	−0.001
			**high**	0.071	−0.003
	**61**	0.076	**low**	0.011	−0.004
			**high**	0.064	−0.013
	**71**	0.077	**low**	0.010	−0.006
			**high**	0.062	−0.027
(b)					
**Diam. 4.89 mm** **Ht. 1.36 mm**	**41**	0.006	**low**	0.001	0.000
			**high**	0.018	0.000
	**61**	0.008	**low**	0.001	−0.003
			**high**	0.016	−0.004
	**71**	0.005	**low**	0.001	−0.004
			**high**	0.015	−0.007
**Diam. 7.33 mm ** **Ht. 2.03 mm**	**41**	0.007	**low**	0.003	−0.001
			**high**	0.035	−0.001
	**61**	0.01	**low**	0.003	−0.003
			**high**	0.033	−0.008
	**71**	0.007	**low**	0.003	−0.005
			**high**	0.030	−0.014
**Diam. 9.77 mm ** **Ht. 2.93 mm**	**41**	0.021	**low**	0.006	−0.001
			**high**	0.052	−0.002
	**61**	0.029	**low**	0.006	−0.003
			**high**	0.049	−0.012
	**71**	0.027	**low**	0.006	−0.006
			**high**	0.046	−0.023
**Diam. 12.20 mm ** **Ht. 4.55 mm**	**41**	0.061	**low**	0.010	−0.001
			**high**	0.068	−0.004
	**61**	0.08	**low**	0.011	−0.004
			**high**	0.062	−0.017
	**71**	0.08	**low**	0.010	−0.007
			**high**	0.060	−0.032

## Data Availability

The original contributions presented in the study are included in the article/Supplementary Material, further inquiries can be directed to the corresponding authors.

## Supplementary Materials

The following supporting information can be downloaded at: https://www.mdpi.com/article/10.3390/cancers16081607/s1, Table S1: Risk of metastatic and non-metastatic death at baseline and after deferring treatment for 4 and 12 months if tumor growth rate is 5% per year; Table S2. Risk of metastatic and non-metastatic death at baseline and after deferring treatment for 4 and 12 months if tumor growth rate is 10% per year; Table S3. Risk of metastatic and non-metastatic death at baseline and after deferring treatment for 4 and 12 months if tumor growth rate is 20% per year; Table S4. Risk of metastatic and non-metastatic death at baseline and after deferring treatment for 4 and 12 months if tumor growth rate is 40% per year; Table S5. Prevalence of monosomy 3 at baseline and after deferring treatment for 4 months, according to tumor growth rate; Table S6. Prevalence of monosomy 3 at baseline and after deferring treatment for 12 months, according to tumor growth rate.
